# Combined speed endurance and endurance exercise amplify the exercise‐induced PGC‐1*α* and PDK4 mRNA response in trained human muscle

**DOI:** 10.14814/phy2.12864

**Published:** 2016-07-25

**Authors:** Casper Skovgaard, Nina Brandt, Henriette Pilegaard, Jens Bangsbo

**Affiliations:** ^1^ Section of Integrated PhysiologyDepartment of Nutrition, Exercise and SportsUniversity of CopenhagenCopenhagenDenmark; ^2^ Team Danmark (Danish elite sports institution)CopenhagenDenmark; ^3^ Department of BiologyUniversity of CopenhagenCopenhagenDenmark

**Keywords:** Combined exercise, endurance exercise, mRNA, muscular response, speed endurance exercise

## Abstract

The aim of this study was to investigate the mRNA response related to mitochondrial biogenesis, metabolism, angiogenesis, and myogenesis in trained human skeletal muscle to speed endurance exercise (*S*), endurance exercise (*E*), and speed endurance followed by endurance exercise (*S* + *E*). Seventeen trained male subjects (maximum oxygen uptake (*V*O_2_‐max): 57.2 ± 3.7 (mean ± SD) mL·min^−1^·kg^−1^) performed *S* (6 × 30 sec all‐out), *E* (60 min ~60% *V*O_2_‐max), and *S* + *E* on a cycle ergometer on separate occasions. Muscle biopsies were obtained at rest and 1, 2, and 3 h after the speed endurance exercise (*S* and *S* + *E*) and at rest, 0, 1, and 2 h after exercise in *E*. In *S* and *S* + *E*, muscle peroxisome proliferator‐activated receptor‐*γ* coactivator‐1 (PGC‐1*α*) and pyruvate dehydrogenase kinase‐4 (PDK4) mRNA were higher (*P* < 0.05) 2 and 3 h after speed endurance exercise than at rest. Muscle PGC‐1*α* and PDK4 mRNA levels were higher (*P* < 0.05) after exercise in *S* + *E* than in *S* and *E*, and higher (*P* < 0.05) in *S* than in *E* after exercise. In *S* and *S* + *E*, muscle vascular endothelial growth factor mRNA was higher (*P* < 0.05) 1 (*S* only), 2 and 3 h after speed endurance exercise than at rest. In *S* + *E*, muscle regulatory factor‐4 and muscle heme oxygenase‐1 mRNA were higher (*P* < 0.05) 1, 2, and 3 h after speed endurance exercise than at rest. In *S*, muscle hexokinase II mRNA was higher (*P* < 0.05) 2 and 3 h after speed endurance exercise than at rest and higher (*P* < 0.05) than in *E* after exercise. These findings suggest that in trained subjects, speed endurance exercise provides a stimulus for muscle mitochondrial biogenesis, substrate regulation, and angiogenesis that is not evident with endurance exercise. These responses are reinforced when speed endurance exercise is followed by endurance exercise.

## Introduction

Exercise training performed at low work rate for a long duration (endurance exercise) results in oxidative adaptations in the muscle and reduced fatigability at a fixed low power output/higher average power output over a fixed distance or time (Gollnick et al. 1973; Green et al. 1995; Gibala et al. 2006; Bogdanis 2012).

Performing speed endurance training (all‐out intermittent 30 sec of exercise at an average of ~90–95% of maximal intensity) and a basic volume of endurance training for 2–9 weeks has been found to improve short‐ and long‐term performance in runners (Bangsbo et al. 2009; Iaia et al. 2009), cyclists (Laursen et al. 2002; Gunnarsson et al. 2013), and soccer players (Thomassen et al. 2010; Christensen et al. 2011; Gunnarsson et al. 2012; Nyberg et al. 2016), but no study has investigated the acute response of combining speed endurance exercise with endurance exercise within the same session.

Peroxisome proliferator‐activated receptor‐*γ* coactivator‐1 (PGC‐1*α*) has been identified as a key regulator of mitochondrial biogenesis and oxidative genes (Puigserver et al. 1998; Handschin and Spiegelman 2006, 2008). A potential explanation for the improved short‐ and long‐term performance found in prior untrained subjects with chronic speed endurance training could be the finding of increased muscle PGC‐1*α* mRNA level as a response to speed endurance exercise (Gibala et al. 2009; Little et al. 2011). However, studies investigating the effects of applying regular speed endurance training in trained subjects do not support this explanation as maximum oxygen uptake (*V*O_2_‐max) and muscle oxidative enzyme activity and protein content remained unchanged. Instead, the improvements may have been caused by improved exercise economy and muscle ion handling (Iaia and Bangsbo 2010).

It was recently demonstrated that the PGC‐1*α* mRNA response was augmented when endurance exercise was followed by resistance exercise versus single‐mode endurance exercise (Wang et al. 2011) indicating that oxidative capacity could be elevated when performing concurrent endurance and resistance exercise compared to single‐mode endurance exercise. Nevertheless, no study has investigated whether the mRNA response to combined speed endurance and endurance exercise would be enhanced compared to single‐mode exercise in trained subjects.

Exercise‐induced angiogenesis has been found to be orchestrated by PGC‐1*α* together with vascular endothelial growth factor (VEGF) (Chinsomboon et al. 2009; Leick et al. 2009) and VEGF mRNA has been reported to increase as a response to supramaximal endurance exercise (Hoier et al. 2013) and endurance exercise (Jensen et al. 2004). Likewise, exercise induced increase in mRNA content of pyruvate dehydrogenase kinase‐4 (PDK4), a protein known to regulate substrate use, has been found to be regulated by changes in PGC‐1*α* mRNA expression (Wende et al. 2005). However, the acute muscle mRNA response to speed endurance versus endurance exercise has not been compared.

Thus, the aims of this study were, in trained subjects: (1) to examine the effect of combined speed endurance and endurance exercise on the mRNA response of proteins related to muscle mitochondrial biogenesis, regulation of substrate use, angiogenesis, and myogenesis in human muscle and compare to the effects of single‐mode exercise, and (2) to compare the mRNA response between single‐mode speed endurance exercise and single‐mode endurance exercise.

We hypothesized that the mRNA response of proteins related to muscle oxidative capacity, substrate regulation, and angiogenesis would be amplified when speed endurance exercise was followed by endurance exercise compared with single‐mode exercise, and that speed endurance exercise would elicit a similar response as endurance exercise in trained subjects.

## Methods

### Subjects

Seventeen trained male subjects with an average age, height, body mass, and *V*O_2_‐max of 23.6 ± 4.2 (mean ± SD) years, 183.4 ± 5.9 cm, 75.5 ± 6.4 kg, and 57.2 ± 3.7 mL·min^−1^·kg^−1^, respectively, took part in the study. After receiving information about the study and the possible risks and discomforts associated with the experimental procedures, all subjects gave their written informed consent to participate. This study conformed to the Code of Ethics of the World Medical Association (Declaration of Helsinki) and was approved by the Ethics Committee of the capital region of Copenhagen (Region Hovedstaden).

### Study design

The study employed a randomized crossover design in which each participant completed three protocols of exercise: (1) speed endurance exercise (*S*), (2) endurance exercise (*E*), and (3) combined speed endurance and endurance exercise (*S* + *E*), separated by minimum a week during which time the participants maintained their habitual activity.

### Preliminary testing and familiarization

Prior to the experimental protocols, subjects performed an incremental cycle test to exhaustion. Testing was performed on a friction‐loaded cycle ergometer (839E; Monark Exercise AB, Varberg, Sweden) with pulmonary *V*O_2_ measured by a breath‐by‐breath gas analyzing system (Oxycon Pro; Viasys Healthcare, Hoechberg, Germany) and heart rate (Polar Team^2^ transmitter; Polar Electro Oy, Kempele, Finland) collected throughout the test. Prior to the experimental protocols, subjects also completed three familiarization sessions during which they performed speed endurance exercise (4, 6, and 6 × 30‐sec all‐out cycling separated by 3 min of active rest).

### Experimental design

**Figure 1:**
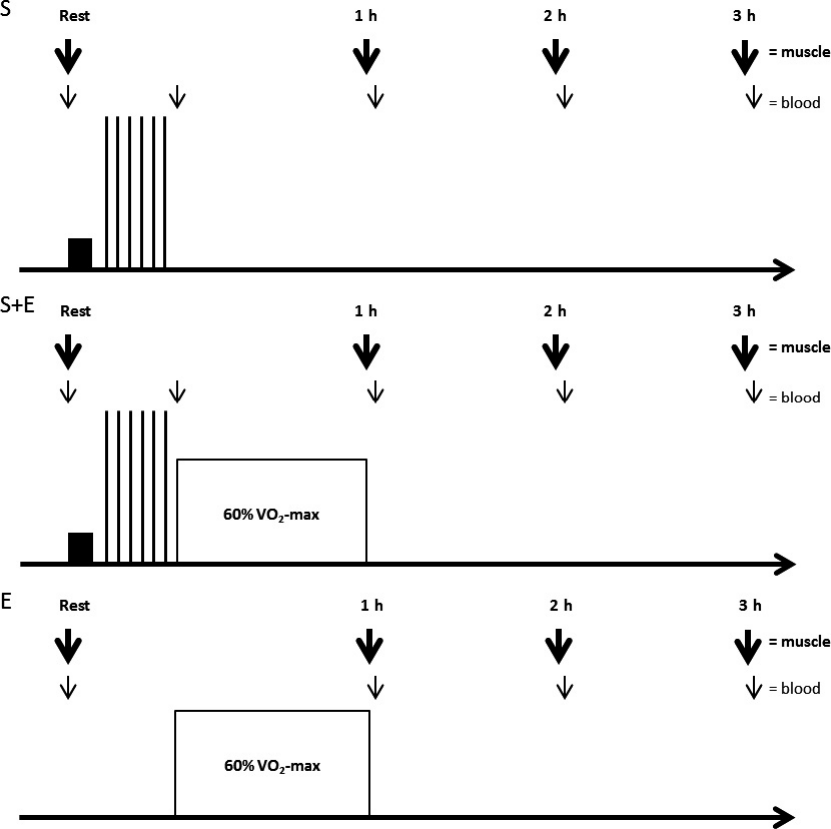
Design of randomized speed endurance exercise (*S*), combined speed endurance and endurance exercise (*S* + *E*), and endurance exercise (*E*) in 17 trained subjects. Post exercise muscle biopsies were aligned after termination of the speed endurance exercise and termed rest, 1, 2, and 3 h for the purpose of clarity.

At least 1 week following the last familiarization session, subjects reported to the laboratory in the morning 2 h after ingesting their typical breakfast, which was replicated for all experimental days. After resting for 15 min, the lateral aspect of the right thigh was anesthetized (1 mL; 20 mg/L lidocaine) and prepared for the extraction of a baseline resting muscle biopsy sample from the vastus lateralis muscle using a Bergstrom needle adapted with suction (Bergstrom 1962). While waiting for the anesthetics to take effect, a catheter was placed in the antecubital vein for collection of blood samples at rest, during exercise, and throughout recovery (Fig. 1). Two muscle biopsies from one incision site on each leg were obtained during each protocol (four muscle biopsies per protocol) and incision sites were separated ~2 cm apart for each protocol. The muscle samples (114 mg wet weight on average) were immediately frozen in liquid N_2_ and stored at −80°C until further analysis.

A standardized 8‐min warm‐up (5 min at 125 W + 3 min at 200 W) preceded experimental protocols *S* and *S* + *E* after which the predefined speed endurance protocol was performed (Monark 939E Analysis Software version 3.0.12.0; Monark Exercise AB). During all experimental protocols, a microprocessor, interfaced with the cycle ergometer, counted flywheel revolutions every second for the duration of the given exercise. The subjects were comfortably seated with feet secured to the pedals by toe clips and heart rate was collected throughout all protocols.

Experimental protocol *S* consisted of 6 × 30‐sec all‐out cycling against a constant resistance of 0.75 × kilograms of body mass (56.6 ± 1.2 [mean ± SE] N) separated by 3 min of active rest (6 N). Subjects were encouraged to pedal as fast as possible from the start and to maintain maximum pedaling speed throughout the 30‐sec periods reaching 148 ± 4 kJ of total work with an average heart rate of 77.3 ± 1.3% HR_max_. *E* consisted of 60 min of cycling at 60% *V*O_2_‐max ([60% *V*O_2_‐max = ((0.6 × *V*O_2_‐max)‐*y*)/*x*] where *x* and *y* make the slope when creating a tendency line based on the *V*O_2_ during the final minute of cycling at 125 and 200 W during the incremental cycle test) corresponding to a power output of 156 ± 5 W amounting to 188 ± 6 kJ of total work with an average heart rate of 77.1 ± 1.1% HR_max_. *S* + *E* was performed as *S* followed by *E* after a 3‐min break amounting to 334 ± 8 kJ of total work with an average heart rate of 79.5 ± 1.6% HR_max_ (78.9 ± 1.6% HR_max_, and 80.2 ± 1.4% HR_max_ *S* and *E*, respectively).

Immediately upon completion of exercise in *S* + *E* and *E*, a muscle biopsy was obtained while subjects were on the cycle ergometer. In *S* + *E* and *E*, subjects were then moved to an adjacent gurney where muscle biopsies were taken 1 and 2 h after exercise (Fig. 1). As muscle biopsies were time‐aligned to the speed endurance exercise, muscle biopsies in *S* were obtained 1, 2, and 3 h after exercise. During recovery from exercise, subjects remained in the laboratory where they rested or worked on a computer and were not allowed to ingest anything except water.

### Muscle analysis

#### RNA isolation, reverse transcription, and real‐time PCR

Total RNA was isolated from 15 to 20 mg muscle tissue (wet weight) by a modified guanidinium thiocyanate–phenol–chloroform extraction method from Chomczynski and Sacchi (1987). Superscript II RNase H‐system and Oligo dT (Invitrogen, Carlsbad, CA) were used to reverse transcribe the mRNA to cDNA as described previously (Pilegaard et al. 2000) except for the use of a TissueLyser (TissueLyser II; Qiagen, Valencia, CA) for homogenization. Quantification of cDNA as a measure of mRNA content of a given gene was performed by real‐time polymerase chain reaction (PCR) using an ABI 7900 sequence‐detection system (Applied Biosystems, Foster City, CA).

Primers and TaqMan probes were designed from human‐specific databases from ensemble (www.ensembl.org/Homo_sapiens/Info/Index) and Primer Express (Applied Biosystems) and are presented in Table 1. Self‐designed TaqMan probes were labeled with 5′‐6‐carboxyfluorescein (FAM) and 3′‐6‐carboxy‐N,N,N′,N′‐tetramethylrhodamine (TAMRA) and obtained from TAG Copenhagen (Copenhagen, Denmark). Single‐stranded DNA content of the cDNA samples was determined using Oligreen reagent (Life Technologies, Carlsbad, CA) as previously described in Lundby et al. (2005).

**Table 1:** Primers and TaqMan probes

mRNA	Forward primer	Reverse primer	TaqMan probe
PGC‐1*α*	5′‐CAAGCCAAACCAACAACTTTATCTCT‐3′	5′‐CACACTTAAGGTGCGTTCAATAGTC‐3′	5′‐AGTCACCAAATGACCCCAAGGGTTCC‐3′
PDK4	5′ TCCACTGCACCAACGCCT 3′	5′ TGGCAAGCCGTAACCAAAA 3′	5′ ATAATTCCCGGAATGCTCCTTTGGCTG 3′
VEGF	5′‐CTTGCTGCTCTACCTCCACCAT‐3′	5′‐ATGATTCTGCCCTCCTCCTTCT‐3′	5′‐AAGTGGTCCCAGGCTGCACCCA‐3′
MRF4	5′ GCTCGTGATAACGGCTAAGGAA 3′	5′ CGATGGAAGAAAGGCATCGA 3′	5′ CAAGTATTGATTCGTCAGCCTCGAGTAGCC 3′
HO‐1	5′ GCCAGCAACAAAGTGCAAGAT 3′	5′ AGTGTAAGGACCCATCGGAGAA 3′	5′ AGAGGGAAGCCCCCACTCAACACC 3′
HK II	5′ TTGTCCGTAACATTCTCATCGATT 3′	5′ TGTCTTGAGCCGCTCTGAGAT 3′	5′ ACCAAGCGTGGACTGCTCTTCCGA 3′

PGC‐1*α*, peroxisome proliferator‐activated receptor‐*γ* coactivator‐1; PDK4, pyruvate dehydrogenase kinase 4; VEGF, vascular endothelial growth factor; MRF4, muscle regulatory factor 4; HO‐1, heme oxygenase‐1; HK II, hexokinase II.

Real‐time PCR was performed (in triplicate) in a total reaction volume of 10 *μ*L using Universal Mastermix with UNG (*Applied Biosystems*, Foster City, CA). The obtained cycle threshold (*C*_t_) values reflecting the initial content of the specific transcript in the samples were converted to a relative amount using standard curves constructed from serial dilution of a pooled sample made from all 204 samples. Target mRNA content (PGC‐1*α*, PDK4, VEGF, muscle regulatory factor 4 [MRF4], heme oxygenase‐1 [HO‐1], and hexokinase II [HK II]; Table 1) was normalized to single‐stranded DNA content to correct for the potential differences in total cDNA content between samples. Single‐stranded DNA content was not affected by exercise and not different between exercise modes as there were no significant differences between exercise mode and/or time points.

### Plasma analyses

For every blood sample collected, a total of ~7 mL blood was drawn in a heparinized 2 mL syringe and a 5 mL syringe. A part of the 2 mL blood sample (~1.5 mL) and the 5 mL sample (split into 2 × 2 mL Eppendorf tubes containing 30 *μ*L ethylenediaminetetraacetic acid) were centrifuged at 20,000*g* for ~2 min and the remaining whole blood from the 2 mL sample (~0.5 mL) was stored on ice for further analyses. After centrifugation, the plasma was transferred into Eppendorf tubes and placed in ice‐cold water until they were stored at −20°C. Plasma samples were subsequently analyzed for adrenaline, noradrenaline, and free fatty acids (FFA) using a Hitachi 912 (Hitachi 912 Automatic Analyzer; Roche Diagnostic, Indianapolis, IN). Whole blood was analyzed for glucose and lactate on an automated blood gas analyzer apparatus (ABL800 Flex; Radiometer Medical, Copenhagen, Denmark).

### Statistics

A two‐way analysis of variance (ANOVA) repeated measures was used to test the effect of exercise mode on mRNA content and blood and plasma variables. Data from mRNA samples exceeding mean ± 2 SD at the given time points were defined as outliers and excluded from the final analysis (a total of 7% of samples were excluded). If a significant main effect was detected (*P* < 0.05), a Student–Newman–Keuls post hoc analysis was used to determine at which time points and/or exercise‐mode mRNA and plasma variables were changed/different and whether interactions were present. Data were presented as mean ± SE unless otherwise stated.

## Results

### Muscle mRNA

#### Peroxisome proliferator‐activated receptor‐*γ* coactivator‐1

**Figure 2:**
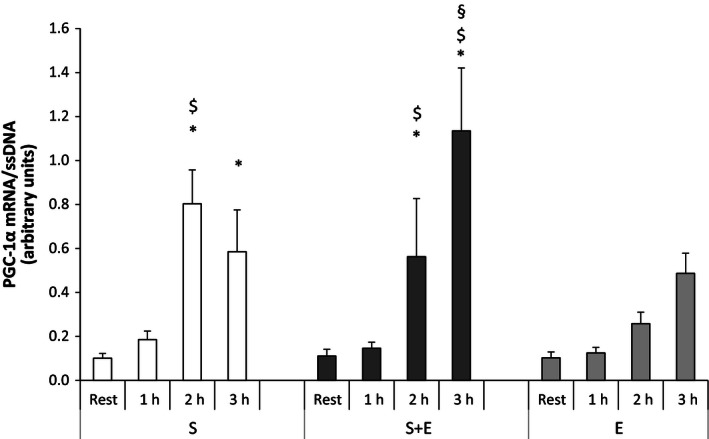
Peroxisome proliferator‐activated receptor‐*γ* coactivator‐1 (PGC‐1*α*) mRNA at rest and during recovery from speed endurance exercise (*S*), combined speed endurance and endurance exercise (*S* + *E*), and endurance exercise (*E*) in 17 trained subjects. Muscle biopsies were obtained at rest, 1, 2, and 3 h after speed endurance exercise in *S* and *S* + *E* and at rest, immediately after exercise, and 1 and 2 h after endurance exercise in *E* (termed rest, 1, 2, and 3 h for the purpose of clarity). PGC‐1*α* mRNA content is normalized to a single‐stranded DNA (ssDNA) and values are given as mean ± SE. *P <* *0.05 different from rest within protocol; ^$^ *P *<* *0.05 different to *E* within time point; ^§^ *P *<* *0.05 different to *S* and *E* within time point.

In *S* + *E*, muscle PGC‐1*α* mRNA was 5‐ and 10‐fold higher (*P* < 0.05) 2 and 3 h after speed endurance exercise than at rest, respectively. In *S*, muscle PGC‐1*α* mRNA was eight and sixfold higher (*P* < 0.05) 2 and 3 h after speed endurance exercise than at rest. No change was found with *E*. Muscle PGC‐1*α* mRNA was higher (*P* < 0.05) in *S* + *E* than *S* 3 h after speed endurance exercise and higher (*P* < 0.05) in *S* + *E* than *E* 1 and 2 h after exercise. The muscle PGC‐1*α* mRNA level 2 h after exercise was higher (*P* < 0.05) in *S* than *E* (Fig. 2).

#### Pyruvate dehydrogenase kinase‐4

**Figure 3:**
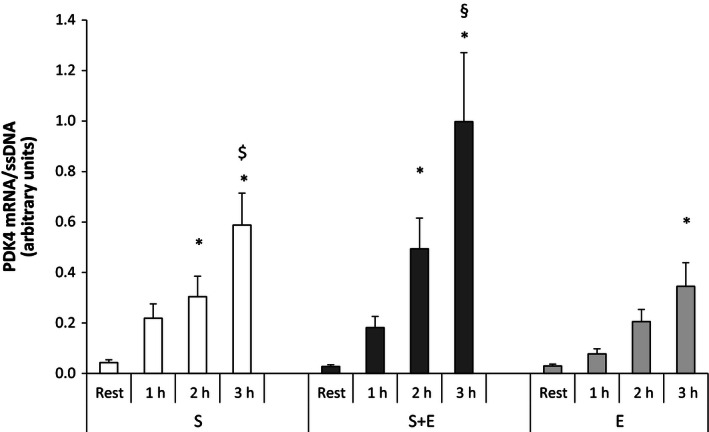
Pyruvate dehydrogenase kinase‐4 (PDK4) mRNA at rest and during recovery from speed endurance exercise (*S*), combined speed endurance and endurance exercise (*S* + *E*), and endurance exercise (*E*) in 17 trained subjects. Muscle biopsies were obtained at rest, 1, 2, and 3 h after speed endurance exercise in *S* and *S* + *E* and at rest, immediately after exercise, and 1 and 2 h after endurance exercise in *E* (termed rest, 1, 2, and 3 h for the purpose of clarity) PDK4 mRNA content is normalized to a single‐stranded DNA (ssDNA) and values are given as mean ± SE. *P <* *0.05 different from rest within protocol. ^$^ *P *<* *0.05 different to *E* within time point; ^§^ *P *<* *0.05 different to *S* and *E* within time point.

In *S* + *E*, muscle PDK4 mRNA was 18‐ and 36‐fold higher (*P* < 0.05) 2 and 3 h after speed endurance exercise than at rest, respectively. In *S*, muscle PDK4 mRNA was 7‐ and 14‐fold higher (*P* < 0.05) 2 and 3 h after speed endurance exercise than at rest, respectively. In *E*, muscle PDK4 mRNA was 12‐fold higher (*P* < 0.05) 3 h after exercise than at rest. The muscle PDK4 mRNA level was higher (*P* < 0.05) in *S* + *E* than *S* and *E* 3 h after speed endurance exercise and higher (*P* < 0.05) in *S* than *E* 3 h after exercise (Fig. 3).

#### Vascular endothelial growth factor

**Figure 4:**
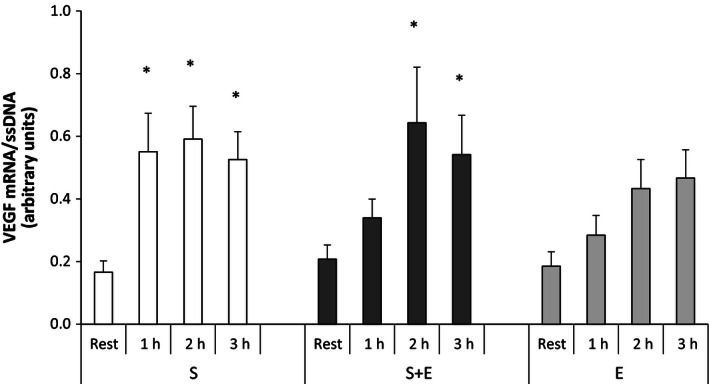
Vascular endothelial growth factor (VEGF) mRNA at rest and during recovery from speed endurance exercise (*S*), combined speed endurance and endurance exercise (*S* + *E*), and endurance exercise (*E*) in 17 trained subjects. Muscle biopsies were obtained at rest, 1, 2, and 3 h after speed endurance exercise in *S* and *S* + *E* and at rest, immediately after exercise, and 1 and 2 h after endurance exercise in *E* (termed rest, 1, 2, and 3 h for the purpose of clarity). VEGF mRNA content is normalized to a single‐stranded DNA (ssDNA) and values are given as mean ± SE. *P <* *0.05 different from rest within protocol.

In *S* + *E*, muscle VEGF mRNA was approximately threefold higher (*P* < 0.05) 2 and 3 h after speed endurance exercise than at rest. In *S*, muscle VEGF mRNA was approximately threefold higher (*P* < 0.05) 1, 2, and 3 h after speed endurance exercise than at rest. No change was found with *E*. No differences were found between exercise modes (Fig. 4).

#### Muscle regulatory factor 4

**Figure 5:**
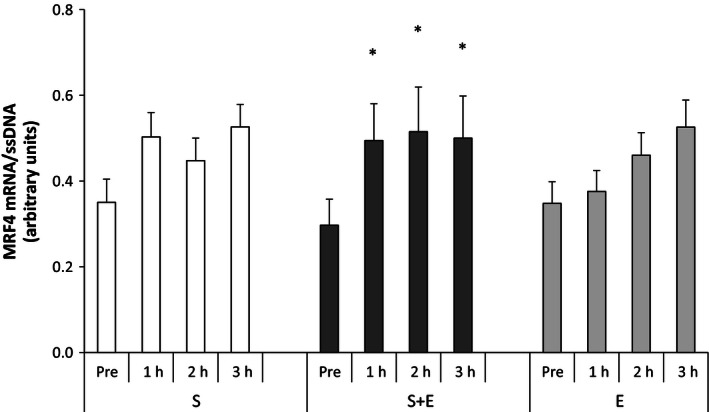
Muscle regulatory factor 4 (MRF4) mRNA at rest and during recovery from speed endurance exercise (*S*), combined speed endurance and endurance exercise (*S* + *E*), and endurance exercise (*E*) in 17 trained subjects. Muscle biopsies were obtained at rest, 1, 2, and 3 h after speed endurance exercise in *S* and *S* + *E* and at rest, immediately after exercise, and 1 and 2 h after endurance exercise in *E* (termed rest, 1, 2, and 3 h for the purpose of clarity). MRF4 mRNA content is normalized to a single‐stranded DNA (ssDNA) and values are given as mean ± SE. *P <* *0.05 different from rest within protocol.

In *S* + *E*, muscle MRF4 mRNA was 1.7‐fold higher (*P* < 0.05) 1, 2, and 3 h after speed endurance exercise than at rest. In *S* and *E*, no changes in muscle MRF4 were found and there were no differences between exercise modes (Fig. 5).

#### Heme oxygenase‐1

**Figure 6:**
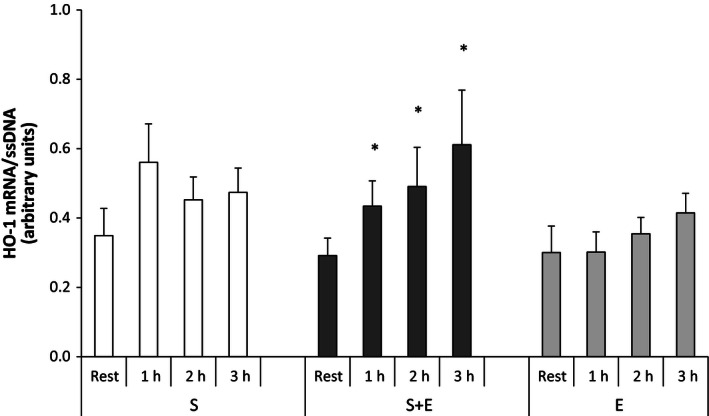
Heme oxygenase‐1 (HO‐1) mRNA at rest and during recovery from speed endurance exercise (*S*), combined speed endurance and endurance exercise (*S* + *E*), and endurance exercise (*E*) in 17 trained subjects. Muscle biopsies were obtained at rest, 1, 2, and 3 h after speed endurance exercise in *S* and *S* + *E* and at rest, immediately after exercise, and 1 and 2 h after endurance exercise in *E* (termed rest, 1, 2, and 3 h for the purpose of clarity). HO‐1 mRNA content is normalized to a single‐stranded DNA (ssDNA) and values are given as mean ± SE. *P <* *0.05 different from rest within protocol.

In *S* + *E*, muscle HO‐1 mRNA was approximately twofold higher (*P* < 0.05) 1, 2, and 3 h after speed endurance exercise than at rest. In *S* and *E*, no changes were observed in muscle HO‐1 mRNA and there were no differences between exercise modes (Fig. 6).

#### Hexokinase II

**Figure 7:**
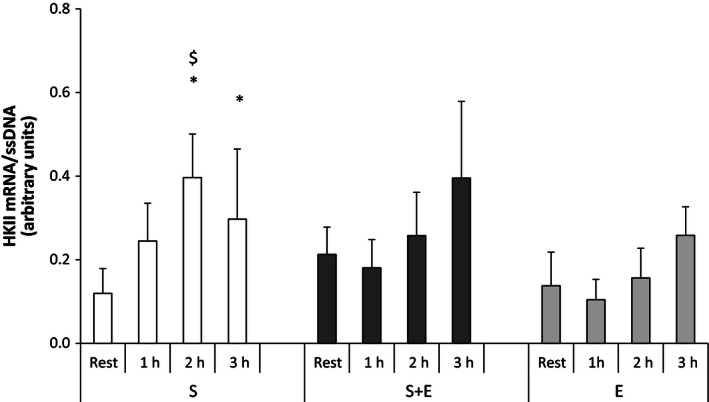
Hexokinase II (HK II) mRNA at rest and during recovery from speed endurance exercise (*S*), combined speed endurance and endurance exercise (*S* + *E*), and endurance exercise (*E*) in 17 trained subjects. Muscle biopsies were obtained at rest, 1, 2, and 3 h after speed endurance exercise in *S* and *S* + *E* and at rest, immediately after exercise, and 1 and 2 h after endurance exercise in *E* (termed rest, 1, 2, and 3 h for the purpose of clarity). HK II mRNA content is normalized to a single‐stranded DNA (ssDNA) and values are given as mean ± SE. *P <* *0.05 different from rest within protocol; ^$^ *P *<* *0.05 different to *E* within time point.

In *S* + *E*, no changes were observed in muscle HK II mRNA. In *S*, muscle HK II mRNA was approximately threefold higher (*P* < 0.05) 2 and 3 h after speed endurance exercise than at rest. No change was found with *E*. The level of muscle HK II mRNA was not different between *S* and *S* + *E*, whereas it was higher (*P* < 0.05) in *S* than in *E* 2 h after exercise (Fig. 7).

### Blood variables

#### Adrenaline and noradrenaline

In *S* + *E*, plasma adrenaline and noradrenaline concentrations were six‐ and eightfold, respectively, higher (*P* < 0.05) 3 min after speed endurance exercise than at rest, and in *S*, the corresponding values were 8‐ and 12‐fold (*P* < 0.05). No changes were found with *E* (Table 2).

**Table 2:** Plasma adrenaline and noradrenaline concentrations before (rest) and 3 min (post) as well as 1 h after speed endurance exercise (in *S* and *S* + *E*) or endurance exercise (in *E*)

	*S*	*S* + *E*	*E*
Adrenaline (mmol/L)			
Rest	0.3 ± 0.1	0.4 ± 0.1	0.6 ± 0.2
Post	2.3 ± 0.4^a^, ^b^	2.5 ± 0.4^a^, ^b^	1.0 ± 0.2
1 h	0.2 ± 0.0^c^	1.3 ± 0.3^a^, ^b^	0.2 ± 0.0
Noradrenaline (mmol/L)			
Rest	2.2 ± 0.3	3.2 ± 0.8	4.5 ± 1.8
Post	26.1 ± 4.1^a^, ^b^	25.4 ± 3.5^a^, ^b^	8.9 ± 1.4
1 h	3.0 ± 0.6^c^	7.7 ± 1.5^b^	1.5 ± 0.2

Values are presented as means ± SE. *S*, speed endurance exercise; *S* + *E*, combined speed endurance and endurance exercise; *E*, endurance exercise.

a Different (*P *<* *0.05) from rest.

b Different (*P *<* *0.05) to *E*.

c Different (*P *<* *0.05) to *S* and *E*.

#### Glucose

In *S* + *E*, plasma glucose increased (*P* < 0.001) from a resting level of 5.0 ± 0.1 to 7.4 ± 0.3 mmol/L 3 min after speed endurance exercise and returned to resting level during the endurance exercise. In *S*, plasma glucose increased (*P* < 0.001) from a resting level of 5.4 ± 0.2 to 7.2 ± 0.3 mmol/L 3 min after exercise. In *E*, plasma glucose did not change. Plasma glucose levels 3 min after speed endurance exercise were similar in *S* + *E* and *S* (Table 3).

**Table 3:** Plasma lactate, glucose, and FFA before (rest) and 3 min (post) as well as 1 and 2 h after speed endurance exercise (in *S* and *S* + *E*) or endurance exercise (in *E*)

	*S*	*S* + *E*	*E*
Lactate (mmol/L)			
Rest	1.3 ± 0.1	1.1 ± 0.1	1.2 ± 0.1
Post	18.8 ± 1.0^a^, ^b^	18.8 ± 0.6^a^, ^b^	2.1 ± 0.2
1 h	4.4 ± 0.4^a^, ^b^, ^c^	2.7 ± 0.2^a^, ^b^	1.0 ± 0.1
2 h	1.8 ± 0.1	1.2 ± 0.1	1.0 ± 0.0
Glucose (mmol/L)			
Rest	5.4 ± 0.2	5.0 ± 0.1	5.3 ± 0.2
Post	7.2 ± 0.3^a^, ^b^	7.4 ± 0.3^a^, ^b^	5.6 ± 0.1
1 h	5.0 ± 0.2	4.8 ± 0.1	5.0 ± 0.1
2 h	5.1 ± 0.1^c^	4.6 ± 0.1	5.1 ± 0.1^c^
FFA (mmol/L)			
Rest	178 ± 31	169 ± 21	164 ± 29
Post	143 ± 17^b^	152 ± 18^b^	713 ± 85^a^
1 h	207 ± 43^c^, ^b^	1038 ± 85^a^, ^b^	715 ± 90^a^
2 h	782 ± 101^a^, ^c^	1353 ± 82^a^, ^b^	790 ± 77^a^

Values are presented as means ± SE. FFA, free fatty acid; *S*, speed endurance exercise; *S* + *E*, combined speed endurance and endurance exercise; *E*, endurance exercise.

a Different (*P *<* *0.05) from rest.

b Different (*P *<* *0.05) to *E*.

c Different (*P *<* *0.05) to *S* and *E*.

#### Free fatty acid

In *S* + *E*, plasma FFA was elevated (*P* < 0.001) approximately six and eightfold 1 and 2 h after speed endurance exercise compared with rest. In *S*, plasma FFA was elevated (*P* < 0.0601) approximately fourfold 2 h after speed endurance exercise compared with rest. In *E*, plasma FFA was elevated (*P* < 0.001) approximately fourfold 3 min after exercise compared with rest and remained at this level 1 and 2 h after exercise. Plasma FFA levels 1 and 2 h after speed endurance exercise were higher (*P* < 0.05) in *S* + *E* than in *S* and *E*. Plasma FFA levels 3 min and 1 h after exercise were lower (*P* < 0.05) in *S* than *E* with no difference 2 h after exercise (Table 3).

#### Lactate

In *S* + *E*, plasma lactate increased (*P* < 0.001) from a resting level of 1.1 ± 0.1 to 18.8 ± 0.6 mmol/L 3 min after speed endurance exercise and decreased during the endurance exercise to 2.7 ± 0.2 mmol/L. In *S*, plasma lactate increased (*P* < 0.001) from a resting level of 1.3 ± 0.1 to 18.8 ± 1.0 mmol/L 3 min after exercise. In *E*, plasma lactate did not change. Plasma lactate levels 3 min after speed endurance exercise were similar in *S* + *E* and *S* (Table 3).

## Discussion

The major findings of this study were that combined speed endurance and endurance exercise resulted in higher post exercise levels of muscle PGC‐1*α* and PDK4 mRNA than single‐mode speed endurance and single‐mode endurance exercise, and only combined speed endurance and endurance exercise increased muscle MRF4 and HO‐1 mRNA levels. In addition, single‐mode speed endurance exercise led to elevated levels of muscle PGC‐1*α*, PDK4, VEGF, and HK II mRNA, which were not observed with single‐mode endurance exercise.

As hypothesized, combined speed endurance and endurance exercise caused higher levels of muscle PGC‐1*α* mRNA than single‐mode endurance exercise. Consistently, Wang et al. (2011) found that resistance exercise (6 × ~11 repetitions of leg press) following endurance exercise in untrained subjects increased muscle PGC‐1*α* mRNA more than endurance exercise only. In this study, plasma lactate concentration peaked at ~19 mmol/L 3 min after the speed endurance exercise, whereas it was ~7 mmol/L 20 min after the concurrent endurance and resistance exercise in the study by Wang et al. (2011). Other studies have found plasma lactate concentrations of ~11 mmol/L immediately after 10 × ~10 repetitions of leg press (Ahtiainen et al. 2015; Apró et al. 2015). Thus, the metabolic demands of the contracting muscles were probably high in both this study and the study by Wang et al. (2011), and the effect on muscle PGC‐1*α* mRNA may have been promoted through AMP‐activated protein kinase (AMPK) activation. Mice studies have suggested that AMPK regulates PGC‐1*α* expression and activity (Jager et al. 2007; McGee and Hargreaves 2010). The level of AMPK is sensitive to energy disturbance (Hardie et al. 2012) and elevated AMPK activity is reported in conditions with prolonged accelerated ATP consumption (Winder et al. 2000; Atherton et al. 2005; McGee and Hargreaves 2010) and 10 × 10 repetitions of leg press (Ahtiainen et al. 2015).

An additional factor may be the high lactate level per se, as lactate has been found to activate mitochondrial biogenesis in cultured cells when incubated with lactate in concentrations observed within muscles during intense exercise (Hashimoto et al. 2007). Nonetheless, this cannot explain the difference in the muscle PGC‐1*α* mRNA level between combined speed endurance and endurance exercise and single‐mode speed endurance exercise as muscle lactate concentrations were similar during the two conditions. Likewise, it has been observed that injections of the *β*2‐adrenoceptor agonist clenbuterol increased PGC‐1*α* mRNA in mouse skeletal muscle (Miura et al. 2007; Chinsomboon et al. 2009), suggesting that adrenaline may play a role in exercise‐induced PGC‐1*α* transcriptional regulation. Thus, the higher levels of adrenaline after the speed endurance exercise with or without subsequent endurance exercise may explain the higher PGC‐1*α* mRNA levels with these exercise‐mode versus single‐mode endurance exercise, but not the difference between speed endurance exercise with or without subsequent endurance exercise.

The above‐mentioned factors (AMPK, lactate, and adrenaline) may also explain why single‐mode speed endurance exercise resulted in higher level of PGC‐1*α* mRNA than single‐mode endurance exercise, despite less work performed. Although not using a randomized crossover design, studies on untrained subjects have reported comparable elevations of PGC‐1*α* mRNA as a response to single‐mode speed endurance exercise and single‐mode endurance exercise (Pilegaard et al. 2003; Russell et al. 2005; Coffey et al. 2006; Gibala et al. 2009; Little et al. 2011). The present finding that muscle PGC‐1*α* mRNA did not change significantly after single‐mode endurance exercise is in contrast to findings of 3‐ to 10‐fold increased levels of PGC‐1*α* mRNA 2–3 h after 60 min of exercise at 60–74% *V*O_2_‐max in untrained and moderately trained subjects (Cluberton et al. 2005; Wang et al. 2011). The lack of increase in muscle PGC‐1*α* mRNA level in this study may be due to the trained subjects being used to endurance exercise, and hence, not sufficiently challenged by 60 min of endurance exercise to induce changes in muscle PGC‐1*α* mRNA. The relative low exercise intensity (60% of *V*O_2_‐max) might also explain the lack of change in PGC‐1*α* mRNA with endurance exercise as PGC‐1*α* mRNA has been shown to increase in an exercise intensity‐dependent manner (Egan et al. 2010; Nordsborg et al. 2010).

This study shows that speed endurance exercise does elevate the level of PGC‐1*α* mRNA in trained subjects. However, this type of exercise may not necessarily lead to muscle mitochondrial biogenesis as performing speed endurance training for a period of 4 weeks did not increase the activity of oxidative enzymes and *V*O_2_‐max in moderately trained runners (Iaia et al. 2008). Likewise, Christensen et al. (2015) and Bangsbo et al. (2009) found unaltered muscle citrate synthase (CS) activity after 7–9 weeks of speed endurance training, and CS protein content even tended to be lowered after the training period in the study by Christensen et al. (2015). These studies reported a marked reduction in training volume, particularly of aerobic moderate intensity training, which may have had a negative influence on muscle mitochondrial biogenesis. In support, the present observation that the PGC‐1*α* mRNA level 3 h after speed endurance exercise was higher after the combined speed endurance and endurance exercise than single‐mode speed endurance exercise indicates that the volume does play a role. On the other hand, speed endurance training combined with separate sessions of aerobic high intensity training led to improved endurance performance in well‐trained cyclists and well‐trained runners (Laursen and Jenkins 2002; Bangsbo et al. 2009) and concurrent speed endurance and heavy resistance training with a basic volume of aerobic training led to better 10‐km performance in a group of moderately trained runners (Skovgaard et al. 2014). Collectively, these studies suggest that endurance performance does improve, but a net synthesis of muscle oxidative enzymes may not occur with speed endurance training, despite an elevated level of muscle PGC‐1*α* mRNA in response to a single bout of speed endurance exercise.

Muscle PDK4 mRNA was elevated with all exercise protocols, but with a more marked increase with combined speed endurance and endurance exercise than single‐mode exercises. Consistently, in untrained subjects, Wang et al. (2011) found a more pronounced (2.2‐fold) muscle PDK4 mRNA response to concurrent endurance and resistance exercise than single‐mode endurance exercise. Nevertheless, studies have shown that PDK4 mRNA increases with endurance exercise (Pilegaard et al. 2000, 2002; Cluberton et al. 2005; Wang et al. 2011), resistance exercise (Yang et al. 2005; Apró et al. 2013), increased muscle PGC‐1*α* expression (Wende et al. 2005), low muscle glycogen content, and high FFA availability (Wu et al. 2001; Pilegaard et al. 2002, 2005; Lane et al. 2015). Muscle glycogen was not measured in this study, but due to the longer exercise exposure during combined speed endurance and endurance exercise than single‐mode exercises, muscle glycogen is likely to have been lower after the combined speed endurance and endurance exercise than the single‐mode exercises. If so, this may have contributed to the higher levels of PGC‐1*α* and PDK4 mRNA as both have shown elevated responses to exercise with low glycogen following endurance (Psilander et al. 2013) and resistance (Camera et al. 2015) exercise. Furthermore, FFA was higher after combined speed endurance and endurance exercise compared to single‐mode speed endurance exercise. Thus, the higher level of muscle PGC‐1*α* mRNA, higher FFA concentration, and possibly lowered glycogen during recovery after combined speed endurance and endurance exercise compared to single‐mode exercise may explain the augmented muscle PDK4 mRNA response compared to single‐mode exercise.

The observation that both combined speed endurance and endurance exercise and single‐mode speed endurance exercise induced a approximately threefold increase in the level of muscle VEGF mRNA during recovery, whereas it was unchanged after endurance exercise in the trained subjects in this study, may suggest that exercise intensity, rather than exercise duration is decisive in evoking a muscle VEGF mRNA response. Nevertheless, 60 min of cycling at an intensity corresponding to 60% of *V*O_2_‐max induced an upregulation of muscle VEGF mRNA in untrained (*V*O_2_‐max: 38 mL·min^−1^·kg^−1^) subjects (Hoier et al. 2012). The finding by Hoier et al. (2012) may be due to the low training status of the subjects. In support of this, 3 h of knee‐extensor exercise has been shown to induce a greater muscle VEGF mRNA response in an untrained than a trained leg (Jensen et al. 2004). Evidently, speed endurance exercise does elevate the level of muscle VEGF mRNA, and the response to endurance exercise seems to depend on the training status of the musculature. As VEGF is the proangiogenic growth factor believed to be the most important in capillary growth (Olfert et al. 2010; Hoier et al. 2012), the present observation suggests that performing speed endurance exercise could potentially lead to increased capillary density.

MRF4 is part of the myogenic regulatory factor family that drive muscle gene expression during myogenesis (Singh and Dilworth 2013) and the level of muscle MRF4 mRNA can increase as a response to both resistance and endurance exercise in untrained (Psilander et al. 2003) and trained subjects (Yang et al. 2005; Harber et al. 2009). The present finding that only combined speed endurance and endurance exercise induced increased muscle MRF4 mRNA level may be caused by the upregulation of muscle PGC‐1*α* mRNA, as PGC‐1*α* has been shown to be associated with accelerated myoblast differentiation (Lin et al. 2014). Thus, it could be speculated that because PGC‐1*α* is a master regulator of mitochondrial biogenesis, the crosstalk between PGC‐1*α* and myoblast differentiation, may coordinate mitochondrial regulation. However, the observation that no change occurred in muscle MRF4 mRNA although the PGC‐1*α* mRNA level increased with single‐mode speed endurance exercise does not support this, and studies are needed to explore this possible relationship.

The HO‐1 isoform is ubiquitous and can increase several fold by stimuli that induce cellular oxidative stress (Hoekstra et al. 2004). The observation that only combined speed endurance and endurance exercise evoked an increase in muscle HO‐1 mRNA may be explained by the higher oxidative stress associated with the greater total work during the combined speed endurance and endurance exercise versus single‐mode exercise. In support, 4 h of endurance cycling at an intensity corresponding to 50–60% of *V*O_2_‐max resulted in a approximately fourfold increase in muscle HO‐1 mRNA in untrained subjects (Pilegaard et al. 2000).

The present finding that only single‐mode speed endurance exercise induced elevated levels of muscle HK II mRNA is in contrast to previous observations showing elevated muscle HK II mRNA in moderately trained subjects (*V*O_2_‐max: 54 mL·min^−1^·kg^−1^) 3 h after combined resistance (8 × 5 80% 1RM) and endurance exercise (30 min cycling at 70% *V*O_2_‐max) (Coffey et al. 2009). Hence, more than 30 min of endurance exercise seems to blunt the increase in HK II mRNA levels found with intense exercise, that is, resistance or speed endurance exercise.

Muscle PGC‐1*α* mRNA does increase in an exercise‐dependent manner (Egan et al. 2010; Nordsborg et al. 2010) and peaks within 2–4 h after exercise (Pilegaard et al. 2003). Based on this, muscle biopsies were aligned after the speed endurance exercise and investigated for up to 3 h of recovery with or without the addition of endurance exercise. For the endurance exercise, muscle biopsies were taken immediately after exercise and up to 2 h of recovery with or without prior speed endurance exercise. This design may not provide a direct comparison of single‐mode speed endurance exercise and single‐mode endurance exercise, as the biopsies after the endurance exercise was obtained 1 h before those collected after the speed endurance exercise. Nevertheless, as mRNA for PGC‐1*α*, VEGF, MRF4, HO‐1, and HK II was not elevated after single‐mode endurance exercise, it is unlikely to affect the interpretations.

In summary, combined speed endurance and endurance exercise increased the levels of muscle PGC‐1*α*, PDK4, VEGF, MRF4, and HO‐1 mRNA content reaching higher levels of muscle PGC‐1*α* and PDK4 mRNA than single‐mode speed endurance and endurance exercise. Single‐mode speed endurance exercise increased muscle PGC‐1*α*, PDK4, and VEGF mRNA levels, which was not found with single‐mode endurance exercise. These findings suggest that speed endurance exercise with or without concomitant endurance exercise provides a stimulus for muscle mitochondrial biogenesis, substrate regulation, and angiogenesis in trained subjects that is not evident with 60 min of endurance exercise. Overall, the response to speed endurance exercise appears to be further enhanced when followed by endurance exercise.

## Conflict of Interest

None declared.
